# Diversity and evolutionary genetics of the three major *Plasmodium vivax* merozoite genes participating in reticulocyte invasion in southern Mexico

**DOI:** 10.1186/s13071-015-1266-7

**Published:** 2015-12-21

**Authors:** Lilia González-Cerón, Rene Cerritos, Jordán Corzo-Mancilla, Frida Santillán

**Affiliations:** Regional Centre for Research in Public Health, National Institute for Public Health, Tapachula, Chiapas 30700 Mexico; Departamento de Medicina Experimental, Facultad de Medicina, Universidad Nacional Autónoma de México, México, DF 04510 México

**Keywords:** *Plasmodium vivax*, southern Mexico, Merozoite, Merozoite surface protein 1, MSP1_42_, Apical membrane antigen 1, AMA1_I-II_, Duffy binding protein, DBP_II_, Genetic diversity, Haplotype network, Recombination, Natural selection

## Abstract

**Background:**

Reported malaria cases in the Americas had been reduced to about one-half million by 2012. To advance towards elimination of this disease, it is necessary to gain insights into how the malaria parasite is evolving, including the emergence, spread and persistence of new haplotypes in affected regions. In here, the genetic diversity of the three major *P. vivax* merozoite genes was analyzed.

**Methods:**

From *P. vivax*-infected blood samples obtained in southern Mexico (SMX) during 2006–2007, nucleotide sequences were achieved for: the 42 kDa carboxyl fragment of the merozoite surface protein-1 (*msp1*_*42*_)*,* domains I-II of the apical membrane antigen-1 (*ama1*_*I-II*_), and domain II of the Duffy binding protein (*dbp*_*II*_). Gene polymorphism was examined and haplotype networks were developed to depict parasite relationships in SMX. Then genetic diversity, recombination and natural selection were analyzed and the degree of differentiation was determined as F_ST_ values.

**Results:**

The diversity of *P. vivax* merozoite genes in SMX was less than that of parasites from other geographic origins, with *dbp*_*II*_ < *ama1*_*I-II*_ < *msp1*_*42*_. *Ama1*_*I-II*_ and *msp1*_*42*_ exposed the more numerous haplotypes exclusive to SMX. While, all *dbp*_*II*_ haplotypes from SMX were separated from one to three mutational steps, the networks of *ama1*_*I-II*_ and *msp1*_*42*_ were more complex; loops and numerous mutational steps were evidenced, likely due to recombination. Sings of local diversification were more evident for *msp1*_*42*_. Sixteen combined haplotypes were determined; one of these haplotypes not detected in 2006 was highly frequent in 2007. The Rm value was higher for *msp1*_*42*_*than for ama1*_*I-II*,_ being insignificant for *dbp*_*II*_. The *dN-dS* value was highly significant for *ama1*_*I-II*_ and lesser so for *dbp*_*II*_. The F_*ST*_ values were higher for *dbp*_*II*_ than *msp1*_*42*_, and very low for *ama1*_*I-II*_.

**Conclusions:**

In SMX, *P. vivax ama1*_*I-II*_, *dbp*_*II*_ and *msp1*_*42*_ demonstrated limited diversity, and exhibited a differentiated parasite population. The results suggest that differential intensities of selective forces are operating on these gene fragments, and probably related to their timing, length of exposure and function during reticulocyte adhesion and invasion. Therefore, these finding are essential for mono and multivalent vaccine development and for epidemiological surveillance.

**Electronic supplementary material:**

The online version of this article (doi:10.1186/s13071-015-1266-7) contains supplementary material, which is available to authorized users.

## Background

*Plasmodium vivax* is the main species causing malaria in the Middle East, Asia, the West Pacific, and Central and South America, where billions of people are at risk [1, 2]. In the Americas, the number of malaria cases gradually declined from 2000 to 2012 due to the improvement and intensification of control measures. In 2012, 469,369 new malaria cases were reported, which represents a 72 % reduction compared to the year 2000 [3, 4]. In 2008, 20,833 cases were reported in Mesoamerica (from Southern Mexico to Panama), of which 11.3 % corresponded to Mexico. Around the world there are countries that have advanced to the pre-elimination phase, while others have reported little decline or even an increase in the number of malaria cases [5, 6]. To contribute to better control efforts and accelerate malaria elimination, more knowledge is needed about the parasites circulating in each region. In this sense, analysis of the evolutionary forces that shape malaria antigen diversity can be useful for monitoring and designing vaccines, describing transmission dynamics, and conducting epidemiological surveillance.

The *Plasmodium* merozoite invades reticulocytes in a few seconds by a series of complex interactions. The merozoite surface protein 1 (MSP1) participates in the initial adhesion to the reticulocyte and the apical membrane antigen 1 (AMA1) into parasite reorientation [7–9]. Then the Duffy binding protein (DBP) irreversible binds at the time of reticulocyte invasion [10]. In *P. vivax* the MSP1 of 200 kb is coded by a gene located in chromosome 7 and is comprised of variable and semi-conserved blocks. This protein is processed into fragments of 83, 30, 38 and 42 kDa [11]. At the time of invasion, the carboxyl fragment of 42 kDa is further processed into two fragments of 33 and 19 kDa (MSP1_33_ and MSP1_19_). Both fragments are immunogenic, capable of inducing antibodies that block merozoite invasion [12]. AMA1 is an antigen of 66 kDa, coded by a gene located in chromosome 9 and secreted by micronemes. The ecto-cytoplasmic region is comprised of three domains (I, II and III) and 16 cysteine residues [13]. The crystal structure of AMA1 shows that domains I and II have a structure similar to the PAN adhesive motive and participate in reticulocyte adhesion, and that domain I is more variable than domain II [8]. DBP is an integral membrane protein of 140 kDa coded by a gene located in chromosome 6 and comprised of seven domains: a signal peptide, two cysteine-rich domains flanking the amino N-cys and carboxyl C-cys (II and VI), three hydrophobic domains (III - V) and one trans-membrane domain (VII) [14]. Domain II (DBP_II_), which comprises 170 amino acids flanked by cisterns 4 and 7 (within residues 291 and 460), binds the Duffy antigen on the reticulocyte surface. This domain also presents high molecular polymorphism, but the precise site of interaction seems to be more conserved [14, 15]. These merozoite proteins induce antibodies capable of blocking reticulocyte invasion. MSP1 seems to be the most immunogenic, evidenced by the fact that a large number of patients developed antibodies against it after the primary blood infection [16, 17]. Apparently, AMA1 is the second most immunogenic [18, 19] and DBP the least [20, 21].

In southern Mexico, previous studies have shown that *P. vivax* comprises a unique genetic population, such as the association of vector compatibility to Pvs25-28 protein polymorphisms [22] and to subpopulations defined by microsatellite markers [23]. By analysis of *msp1* block icb5-6 from this region, divergent lineages of Sal 1 or Belem strains were distinguished [24]. To gain further insights into the evolutionary genetics of *P. vivax* merozoite vaccine candidates, the genetic diversity of *ama1*_*I-II*_, *dbp*_*II*_ and *msp1*_*42*_ fragments was analyzed in parasites from southern Mexico and compared to other geographic origins.

## Methods

This study was approved by the Ethics Committee of the National Institute of Public Health in Mexico (INSP, according to the initials in Spanish). Informed consent was obtained from all adult patients and the guardians of minors.

The specific area of southern Mexico herein studied is its Pacific coastal plain, defined as Jurisdiction VII of the State of Chiapas. This jurisdiction comprises 4644.07 km^2^ and during the period of the present study (2006–2007) had a population of 710,716 inhabitants. Its altitude ranges from sea level to mountainous regions of about 4000 m above sea level, and has a great climactic and biological diversity. In the State of Chiapas, malaria transmission has been persistent but fluctuating. Since the 1980’s, when over 20, 000 cases were reported per year, malaria transmission has been declining. In the late 1990’s, there were about 1000 cases reported per year [25]. In Jurisdiction VII of the Chiapas State only 167 cases were reported in 2005. After hurricane Stan hit the region in October of 2005, the anti-malarial brigades did not have access to the affected areas for at least four months. As a result, the number of malaria cases increased in 2006 to 646 and in 2007 to 644 (data from the local malaria control program, Sanitary Jurisdiction VII).

### *P. vivax* blood samples

During 2006 and 2007, malaria symptomatic patients living in Jurisdiction VII and diagnosed with *P. vivax* infection by microscopy were invited to donate 5 ml of venous blood. The DNA from infected blood samples was extracted using a commercially available QIAamp DNA blood minikit (Qiagen, USA), following the manufacturer’s instructions. From 200 μL of heparinized infected blood, two hundred microliters of soluble DNA were obtained.

### PCR amplification and DNA sequencing

The *msp1*_*42*_ gene fragment of approximately 1.2 kb was amplified using primers *msp1-*5´AAGCTTAGAGGACTACGACAAAG -3' and *msp1-*3´GTCGAAGGATTCGAACGAGCTCG 5'. The PCR was prepared as follows: 60 mM de Tris-SO_4_, 18 mM ammonium sulphate, 2 mM of MgSO_4_, 0.2 mM of dNTPs, 0.4 μM of each primer, 1U Platinum Taq DNA polymerase High Fidelity (Invitrogen, Carlsbad, CA) and 1–4 μL of genomic DNA, for a final PCR volume of 50 μL. The reaction was run at 95 °C for 5 min, followed by 35 cycles of 95 °C for 60 s, 60 °C for 60 s and 72 °C for 75 s, and a final extension at 72 °C for 10 min.

The *ama1*_*I-II*_ gene fragment of approximately 1.1 kb was amplified using primers *ama1f-*5'-TCCAGCTGGAAGATGTCCTG-3' and *ama1r-*5'-CCGCCCTTTTCTCTACACAG-3'. The PCR was prepared as aforementioned and the reaction was run at 95 °C for 5 min, followed by 35 cycles of 95 °C for 60 s, 61 °C for 60 s and 72 °C for 75 s, and a final extension at 72 °C for 10 min.

The *dbp*_*II*_ gene fragment of approximately 1.2 kb was amplified by polymerase chain reaction (PCR) using the following primers: *dbpf*-5' GATAAAACTGGGGAGGAAAAAGAT 3' and *dbpr*-5' CTTATCGGATTTGAATTGGTGGC 3'. The PCR was prepared as aforementioned and the reaction was run at 94 °C for 3 min, followed by 35 cycles of 94 °C for 40 s, 58 °C for 40 s and 72 °C for 1.5 min, and a final extension at 72 °C for 5 min.

The amplified products were examined in agarose gels at 1 % and stained with 0.2 μg/ml ethidium bromide using a Midicell primo electrophoresis chamber (Thermo EC330, New York, USA). The 100 bp ladder was used as a molecular marker (Invitrogen Corporation, Carlsbad, CA). The gel image was obtained in a photo documentation UV system (LMS-20E Bio-Doc-it, Upland, CA).

The amplified products, estimated by visual observation, were purified using a MinElute PCR purification kit (Qiagen, Valencia, CA), following the manufacturer’s instructions. The purified products were sequenced using forward and reverse primers for merozoite genes in a Taq FS Dye Terminator Cycle Fluorescence-Based Sequencer (Perkin Elmer/Applied Biosystems Model 3730) at the Biotechnology Institute of the National Autonomous University of Mexico at Cuernavaca (Morelos, Mexico). The quality of pherograms with the forward and reverse nucleotide sequences was verified manually and by using Bioedit v7.1.3 software. Consensus sequences were obtained for each gene fragment and were submitted to the NCBI GenBank [accession numbers: KP759780–KP759884].

### Data analysis

The consensus sequences were aligned to the corresponding reference gene strains Sal 1 and Belem, respectively, as follows: for *dbp*_*II*_, XM_001608337.1 and EU395587; for *ama1*_*I-II*_, XM_001615397 and EU395595; for *msp1*_*42*_, XM_001614792.1 and AF435594. DNA polymorphism was analyzed for the three gene fragments. The number of segregating sites (polymorphic) (S), number of mutations or nucleotide changes (M), number of haplotypes (H), haplotype diversity (Hd), nucleotide and genetic diversity (Pi; π, Theta; θ), and their corresponding standard deviations (SD) were calculated with DnaSP v5.1 software [26]. Haplotype frequencies for each gene fragment and for combined haplotypes were examined over time.

To explore the genetic relationship between the haplotypes of each merozoite gene, and how these haplotypes show up in the parasite isolates, a minimum spanning haplotype network was constructed for each gene fragment using TCS 1.21 [27] and Median Joining Network in PopArt 1.7 [28].

To determine how selective forces influence the haplotype relationship, the minimum number of recombination events (Rm) was estimated by the four-gamete test [29]. *D’* and *R*^*2*^ indexes of linkage disequilibrium were determined by non-random association between nucleotide variants within the gene fragments using DnaSP. To test the neutral theory of evolution, Tajima’s D values were calculated. Positive values correspond to positive or balancing selection, whereas negative values correspond to negative or purifying selection [30]. In coding regions, an excess of non-synonymous relative to synonymous changes is a clear signal of positive selection. Different levels of positive selection have been reported in other geographic sites for *msp1*_*42*_ [31–33], *ama1*_*I-II*_ [13, 34] and *dbp*_*II*_ [35, 36]. Hence, the *dN-dS* difference between the rate of the average number of non-synonymous (dN) and synonymous (dS) nucleotide changes was calculated for each gene fragment of the distinct parasite groups by using the alternate hypothesis (HA: *dN > dS*). For this purpose, we used Nei-Gojobori’s method [37] and bootstrap variance estimation with 1000 replicates for the Z-test of selection (*p < 0.05*) in MEGA v6.0 software [38].

Finally, Wright’s fixation statistics (F_ST_) [39] indexes were calculated to determine the degree of differentiation of *P. vivax* in southern Mexico based on each merozoite gene (*dbp*_*II*_, *msp1*_*42*_ and *ama1*_*I-II*_), using DNA sequences of parasites from different geographic origins, by using the model of two parameters of Kimura 2P in DnaSP [26, 40]. F_ST_ values range from 0 to 1, high values indicate considerable degree of differentiation between parasite populations.

Homologous DNA sequences from other geographic sites were obtained from the NCBI GenBank: *Msp1*_*42*_. For South Korea (SK), *n* = 200: JQ446312-JQ446322 [41], HQ171934-HQ171941 [42], and AF435635-AF435638 [11]. For Turkey (TUR), *n* = 30: AB564559-AB564588 [43]. For Thailand (THL), *n* = 93: AF435595-AF435615 [11], GQ890917-GQ890974 [44], AF199393- AF199404, and AF199408- AF199410 [45]. For Singapore (SNG), *n* = 50: GU971656-GU971705 [46]. For India-Bangladesh (IND-BNG), *n* = 35: EU430452-EU430479 [31], KF612323 [47], AF435639, and AF435616-AF435620 [11]. For Brazil (BRZ), *n* = 11: AF435622-25, 27, 29, 30, 31,[11] and AF199405-7 [45]. For Sri Lanka (SLK), *n* = 106: AJ292349-AJ292359 and GU175174-GU175268 [32]. For Myanmar (MYN), *n* = 28: JX490129-JX490156 (Zhou and Chen, unpublished). For Cambodia (CAM), *n* = 44: JX461286-JX461310, JX461312, JX461313, and JX461315- JX461332 [48]. *Ama1*_*I-II*_: For THL, *n* = 231: FJ784891-FJ785121 [49]. For Venezuela (VNZ), *n* = 73: EU346015-EU346087 [34]. For IND, *n* = 56: EF025187-EF025197 [50] and EU282774-EU282822 [51]. For SLK, *n* = 23: EF218679-EF218701 [13]. For Iran (IRN), *n* = 83: JX624732-35 [52], KF422636-681, and KF422636-KF422681 [53]. For Papua New Guinea (PNG), *n* = 102: KC702402-KC702503 [54]. *Dbp*_*II*_: For MYN, *n* = 54: JN255576-JN255587 [55]. For SK *n* = 111: JN989472-JN989484 [56], AF215737-AF215738 [57], AF220657-AF220663, AF220668 [58], DQ156522, DQ156523, and DQ156515 (Lim and Ayala, unpublished). For PNG, *n* = 201: AF289480-AF289483, AF289635-AF289640, AF289642-AF289645, AF289647-AF289653, AF291096 [59], AF469515-AF469602 [60], AY970837-AY970925 [61], and DQ156519 (Lim and Ayala, unpublished). For THL, *n* = 30: EF219451, EF368159-EF368180, EF379127, EF379128, and EF379130-EF379134 [35]. For BRZ, *n* = 122: EU812839-EU812960 [62]. For IND, *n* = 95: DQ156514, DQ156516 (Lim and Ayala, unpublished), FJ491142-FJ491144, FJ491146-FJ491161, FJ491163-FJ491169, FJ491171-FJ491173, FJ491175-FJ491218, FJ491221-FJ491234, and FJ491236-FJ491241 (Prajapati and Joshi, unpublished). For Colombia (COL), *n* = 17: U50575-U50591 [63]. For SLK, *n* = 100: GU143914-GU144013 [64]. For IRN, *n* = 130: EU860430-EU86038 [65], KF318358, KF318359 KF791921, and KF791923-KF791926 [66].

## Results

### Genetic polymorphism of *P. vivax* merozoite genes in SMX

From 35 *P. vivax* isolates (2006, *n* = 17; 2007, *n* = 18), gene fragments *dbp*_*II*_, *ama1*_*I-II*_ and *msp1*_*42*_ were amplified and the consensus nucleotide sequence obtained. Although several *P. vivax* isolates had codons or nucleotide fragments that resembled either Sal 1 or Belem, none was identical to either of these sequences. The *msp1*_*42*_ sequence was more polymorphic than that of *dbp*_*II*_ or *ama1*_*I-II*_ (Table 1).

**Table 1:** Comparison of *P. vivax* merozoite gene diversity among isolates from southern Mexico, 2006–2007

Parameter	Gene fragment		
	*msp1*_*42*_	*ama1*_*I-II*_	*dbp*_*II*_
bp	1071	885	1104
^1^n, polymorphic sites	52	14	14
n, nucleotide changes	59	14	14
^2^π (SD)	0.01716 (0.00179)	0.00745 (0.00039)	0.00304 (0.00062)
^3^Θw (SD; nr, fr)	0.01178 (0.00381, 0.00163)	0.00384 (0.00150, 0.00103)	0.00308 (0.00120, 0.00082)
n, haplotypes	9	7	8
^4^Hd (SD)	0.783 (0.051)	0.723 (0.057)	0.556 (0.094)
Tajima’s D test (*P* value)	1.0408 (>0.10)	3.0416 (<0.01)	- 0.0459 (>0.10)

bp, base pairs; ^1^Number; ^2^Nucleotide diversity (all nucleotide sites); ^3^nr, no recombination, and fr, free recombination; ^4^Haplotype diversity; SD, standard deviation

The *msp1*_*42*_ gene fragment of 1072 bp (codons 1353–1710) had 52 polymorphic sites, in which 59 nucleotide changes were detected. All nucleotide changes were detected in subfragment *msp1*_*33*_ (nucleotides 4060–4875), and five of these were synonymous. Subfragment *msp1*_*19*_ was conserved (Table 1; Additional file 1). Nine haplotypes were resolved and the haplotype diversity (Hd) was higher than that found for *dbp*_*II*_.

Fourteen polymorphic sites and seven haplotypes were detected among the 35 isolates for the *ama1*_*I-II*_ gene fragment of 885 bp (Table 1). Domain I had 11 nucleotide changes (within codons 125–243) and domain II three (within codons 244–460). Compared to the Sal 1 sequence, three additional polymorphisms were detected, with one synonymous change at codon 137 Pro (cca → ccc), and two nonsynonymous changes at codons 145 Glu/Ala (gaa → gca) and 277 Glu/Lys (gag → aag). These variant codons resembled the sequence of the Belem strain (Additional file 2).

A gene fragment of 1,104 pb was obtained for *dbp*_*II*_. Fourteen polymorphic sites and mutations were detected within codons 191–558. The only synonymous change, at codon 530 (aac → aat), was found in two isolates. Most of the diversity indexes were lower than those estimated for *ama1*_*I-II*_ and *msp1*_*42*_ (Table 1; Additional file 3).

### Temporal distribution of single and combined haplotypes

**Fig. 1:**
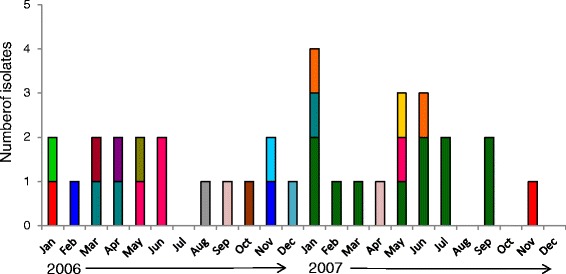
Monthly distribution of *P. vivax dbp*_*II*_ *- ama1*_*I-II*_ - *msp1*_*42*_ combined haplotypes in southern Mexico, 2006–2007. Each haplotype is indicated by a different color. Of 16 combined haplotypes, 13 were detected in 2006 and seven in 2007. Four were common to both years, while nine were exclusive to 2006 and three to 2007. The most frequent combined haplotype (in green) was only detected during 2007

*P. vivax* haplotypes were detected at a different frequency over time (Fig. 1; Additional file 4). The highest number of haplotypes for all three gene fragments (*dbp*_*II*_*, ama1*_*I-II*_*and msp1*_*42*_) was found in 2006, when all eight *dbp*_*II*_ and seven of the *ama1*_*I-II*_ haplotypes were detected. Meanwhile, only three *dbp*_*II*_ and four *ama1*_*I-II*_ haplotypes were identified in 2007. For *msp1*_*42*,_ eight of nine total haplotypes were detected in 2006. In addition, of 16 combined haplotypes (*dbp*_*II*_*-ama1*_*I-II*_*-msp1*_*42*_), 13 were detected in 2006 and seven in 2007 (four of them were found in both years). For the three haplotypes exclusively detected in 2007, haplotype A was significantly more frequent (59 %) than the others (*p* = 0.00024) and was detected from January to September.

### Haplotype relationships for different merozoite genes and the haplotype configuration for *P. vivax* isolates in SMX

**Fig. 2:**
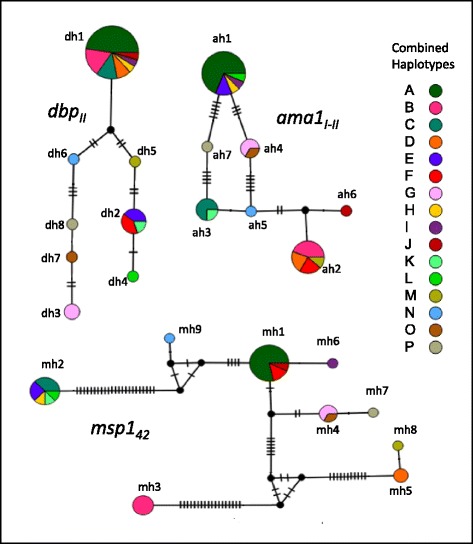
Haplotype networks of *P. vivax* genes encoding merozoite surface proteins in southern Mexico, 2006–2007. The haplotype network for each gene marker is shown. Each circle represents one haplotype and the circle size indicates frequency. The mutational steps are indicated by the short marks crossing the connection lines. Each color indicates a particular combined haplotype (*dbp*_*II-*_ *ama1*_*I-II*_-*msp1*_*4*_, *n* = 16; coded by letters A → P). For example, isolates in dark green had the combined haplotype dh1-ah1-mh1, which was highly frequent and only detected in 2007. Meanwhile, isolates in intense pink and orange had a very different configuration (dh1, ah2 and mh3 or mh5), and haplotypes G, N, O and P had a distinctive and unique configuration

Each gene fragment produced a haplotype network with a different pattern (Fig. 2). 1) The SMX *dbp*_*II*_ network was the simplest; the haplotypes were separated by one to three mutational steps. From a highly frequent haplotype (dh1; 65.7 %), two branches emerged, and the most distant haplotypes (dh3 or dh4) were separated in opposite directions by eight and eleven mutational steps, respectively. Rather than a network, a straight-line pattern was observed. 2) For SMX *ama1*_*I-II*_*,* the highly frequent haplotype (ah1; 45.7 %) and another four formed a loop, with 3 - 5 mutational steps in between. From haplotype ah5 there was a branch connecting more divergent haplotypes, ah2 and ah6. 3) The SMX *msp1*_*42*_ network was more complex than that of *ama1*_*I-II*_. Several haplotypes (mh2, mh3 and mh5) were apparently separated from the most frequent haplotype (mh1; 40 %) by numerous mutational steps. The triangle-like structures suggest the presence of different selective forces. Sign of  local divergence was detected in all three gene fragments, and it was more apparent in *msp1*_*42*_. Haplotypes mh6, mh7 and mh8 seemed to have appeared by a mutation from other more frequent haplotypes. Haplotype mh6 had a nonsynonymous change exclusive to SMX at codon 1625 Lys/Glu (aag → gag). A total of 16 different *dbp*_*II*_ - *ama1*_*I-II*_*- msp1*_*42*_ haplotype arrangements were displayed (indicated by colors in Fig. 2). That is, *P. vivax* isolates with haplotype *dbp*_*II*_ dh1 could be accompanied by different and highly divergent *ama1*_*I-II*_ and *msp1*_*42*_ haplotypes; for example, ah1, ah2, ah3 or ah6, and mh2, mh3 or mh5 and so on.

#### Recombination and linkage disequilibrium

**Fig. 3:**
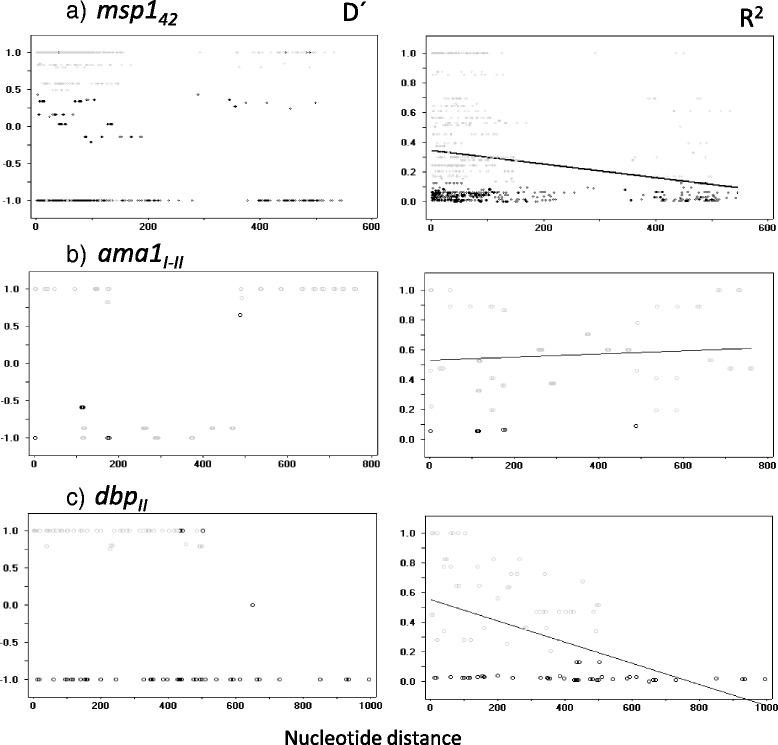
Linkage disequilibrium (D’ and R^2^ indexes) in *P. vivax* merozoite gene fragments: ***a***) *msp1*_*42*_ *,* **b**) *ama1*_*I-II*_ and **c**) *dbp*_*II*_. Significant *D’* values for all gene fragments were mostly polarized to the upper and lower range (from −1.0 to 1.0), except in *msp1*_*42*_. R^2^ values were high and diminished with nucleotide distance in *dbp*_*II*_ and *msp1*_*42*_, while remaining high in *ama1*_*I-II*_. Circles in grey indicate a significant non-random association between nucleotide variants at different polymorphic sites, according to Fisher’s exact test at a 95 % confidence level

Rm values were higher for *msp1*_*42*_ than *ama1*_*I-II*_ and *dbp*_*II*_ (Table 2). All three gene markers were under linkage disequilibrium, with *D’* values polarized to the upper and lower sides of the graph (−1.0 or 1.0). For *msp1*_*42*_, a set of values ranged from 0.5 to −0.25, and the *R*^*2*^ regression line started from ~0.35 (the lowest value for the three gene fragments) and decreased slowly with nucleotide distance. The graph shows the significant values of Fisher’s exact test; 203 significant comparisons were detected using the Bonferroni correction; *R*^*2*^ values were above 0.5. For *dbp*_*II*_, *R*^*2*^ regression started at ~0.58 and sharply decreased to zero; with the Bonferroni correction only 18 comparisons were significant; *R*^*2*^ values were above 0.45. For *ama1*_*I-II*_, on the other hand, the *R*^*2*^ regression line showed a subtle increase across the nucleotide distance; 74 significant comparisons were found with the Bonferroni correction; *R*^*2*^ values were above 0.378 (Fig. 3).

**Table 2:** Natural selection and recombination of *P. vivax* genes encoding merozoite proteins

Merozoite gene fragment	Geographic origin	N	*dN-dS*				Recombination Rm
			*Synonymous	*Non-synonymous	Z-test	*p*	
***msp1***_***42***_	SMX	35	6	34	1.276	*0.102*	9
	Global	632	21	88	2.755	*0.007*	22
***ama1***_***I-II***_	SMX	35	1	13	2.157	*0.016*	3
	Global	607	15	36	1.562	*0.121*	17
***dbp***_***II***_	SMX	35	0	10	3.161	*0.001*	1
	Global	896	34	125	2.587	*0.005*	18

SMX, southern Mexico; N, number of sequences; Rm, minimal number of recombination events. *msp1*_*42*_, 981pb; *ama1*_*I-II*_, 780pb; *dbp*_*II*_, 663pb. *Codons with multiple evolutionary paths were excluded from the analysis

#### Natural selection

Tajima’s D value was positive for *msp1*_*42*_ and *ama1*_*I-II*_, although statistical significance existed only for *ama1*_*I-II*_. The value for *dbp*_*II*_ was near neutrality (Table 1). For all gene fragments, non-synonymous nucleotide changes were more numerous than synonymous ones, and *dN-dS* values were consistently above 1.0 (Table 2). Unlike *msp1*_*42*_, highly significant *dN-dS* values were detected for *ama1*_*I-II*_ and *dbp*_*II*_.

#### Genetic comparison of *P. vivax* merozoite genes from SMX to other geographic sites

In SMX, the highest nucleotide diversity (π) was for *msp1*_*42*_*,* followed by *ama1*_*I-II*_ and finally *dbp*_*II*_ (Table 1). The genetic and nucleotide diversity for SMX *msp1*_*42*_ was lower than that found in all other sites (e.g., BRZ, IND and MYN) except TUR and SK (Additional file 5a). Similarly, the nucleotide diversity of SMX *ama1*_*I-II*_ was lower than that for all other locations except VNZ (Additional file 5b), while the SMX *dbp*_*II*_ fragment showed the lowest diversity of all locations (Additional file 5c). In contrast, Asian parasites were the most diverse for these three gene markers.

A sample of 35 isolates is acceptable for diversity analysis [67]. There were similar or smaller samples than SMX and from high transmission regions and the diversity results were as high as expected (e.g. BRZ, IND and MYN for *msp1*_*42*_; THL, IND and SLK for *ama1*_*I-II*_; COL and THL for *dbp*_*II*_) (Additional file 5). There were differences in the number of exclusive haplotypes detected in each gene fragment; *msp1*_*42*_ and *ama1*_*I-II*_ > *dbp*_*II*_ (Additional file 6). For SMX *dbp*_*II*_, many haplotypes (including the very frequent ones) were shared with BRZ and sites outside the Americas. Two *ama1*_*I-II*_ haplotypes were only detected in VNZ (ah3 and ah4). Meanwhile, the most abundant *msp1*_*42*_ (mh1) haplotype was also detected in BRZ, TUR and SK, and mh3 was also found in THL. Haplotype mh6 was only detected in one isolate and had one nonsynonymous change exclusive for SMX.

The Rm value of nine found for SMX *msp1*_*42*_ was slightly lower than the range of 10 to 13 calculated for other locations (SLK was the exception, with a value of 23; Additional file 7). Similarly, Rm values for *ama1*_*I-II*_ and *dbp*_*II*_ (three and one, respectively) were significantly lower in SMX than those found for other locations (ranging from six to eleven, *p = 0.0008,* and from five to ten, *p = 0.0003*, respectively; Additional file 7).

Similar to SMX, a significant positive *dN-dS* was detected in *P. vivax ama1*_*I-II*_ from VNZ, PNG and SLK, but this parameter was not significant for IRN or IND parasites. Globally, positive selection was not significant (Table 2). However, this lack of significance may be due to the low value (0.368; *p* = 0.357) and large sample size (231 isolates) of THL parasites (Additional file 7). Indeed, the global analysis without THL resulted in a significantly positive *dN-dS* value (2.291, *p = 0.012*). For *msp1*_*42*_ of *P. vivax* parasites, this parameter had a significant positive value in most countries, except SMX, TUR and BRZ, and to a lesser extent SLK (Additional file 7).

### Genetic differentiation of *P. vivax* based on merozoite gene markers

F_ST_ values for *P. vivax* merozoite genes varied between intra and inter-continental regions, and there was no relation to geographic origin (Table 3). For *msp1*_*42*_, F_ST_ values were from 0.0918 to 0.2859 between SMX parasites and those of other geographic origins. In fact, the lowest values were between the parasites of SMX and those of BRZ, IND-BNG and SLK. Interestingly, the highest F_ST_ values were observed (0.2977–0.5069) between TUR and other geographic sites, except between TUR and SMX (the lowest value, 0.1332).

**Table 3:** F_*ST*_ indexes for *P. vivax* based on merozoite genes; values between southern Mexico and other geographic sites are in bold

^a^ *msp1*_*42*_									
	**SMX**	BRZ	TUR	SK	SNG	THL	CAM	IND-BAN	MYN
BRZ	**0.1065**								
TUR	**0.1332**	0.3554							
SK	**0.2859**	0.2229	0.5069						
SNG	**0.2370**	0.2754	0.4064	0.3221					
THL	**0.1817**	0.1129	0.3803	0.2760	0.1007				
CAM	**0.1710**	0.0806	0.3637	0.2279	0.1366	0.0093			
IND-BAN	**0.0978**	0.0427	0.3074	0.1139	0.1273	0.0476	0.0266		
MYN	**0.1754**	0.0968	0.3596	0.2189	0.0966	−0.0081	−0.0042	0.0192	
SLK	**0.0918**	0.0377	0.2977	0.2099	0.1531	0.0365	0.0374	0.0119	0.0267
^b^ *ama1*_*I-II*_									
	**SMX**	VNZ	IRN	IND	SLK	THL			
VNZ	**0.1881**								
IRN	**0.0799**	0.1220							
IND	**0.1080**	0.1395	0.0066						
SLK	**0.1402**	0.1682	0.0830	0.0740					
THL	**0.1983**	0.1580	0.1133	0.0934	0.0850				
PNG	**0.2374**	0.2859	0.1194	0.0803	0.1738	0.1993			
^c^ *dbp*_*II*_									
	**SMX**	COL	BRZ	IRN	IND	SLK	SK	THL	MYN
COL	**0.3157**								
BRZ	**0.2269**	0.1386							
IRN	**0.2255**	0.1812	0.0487						
IND	**0.2476**	0.1640	0.0132	0.0668					
SLK	**0.2652**	0.1755	0.0303	0.0923	0.0260				
SK	**0.2601**	0.2992	0.1327	0.1895	0.1222	0.1263			
THL	**0.1767**	0.1922	0.0616	0.1414	0.0764	0.0873	0.1369		
MYN	**0.2040**	0.2364	0.0840	0.1484	0.0810	0.0982	0.1286	0.0016	
PNG	**0.2799**	0.2057	0.1260	0.1745	0.1483	0.1376	0.2252	0.1205	0.1452

SMX, southern Mexico; IRN, Iran; IND, India; BRZ, Brazil; COL, Colombia; PNG, Papua New Guinea; SLK, Sri Lanka; THL, Thailand; SK, South Korea; MYN, Myanmar; VNZ, Venezuela; SNG, Singapore; BNG, Bangladesh; TUR, Turkey; CAM, Cambodia

The values for *ama1*_*I-II*_ were from 0.0799 to 0.2374 between SMX parasites and those of other geographic origins. The lowest value was between SMX and IRN, while the highest values were found when comparing SMX or VNZ to PNG. SMX vs THL or VNZ showed similar values (0.1983 vs 0.1881, respectively). It is notable that *P. vivax dbp*_*II*_ produced high F_ST_ values between SMX and many other locations (0.1767–0.3157). Strikingly, the highest value was between SMX and COL, which was greater than that found between SMX and PNG or SK. The latter results are in agreement with the values for *ama1*_*I-II*_ (between SMX and PNG). The F_ST_ value between SMX and THL was similar for the three merozoite genes (0.1767–0.1983). Regarding SMX vs IND-SLK, there were low F_ST_ values for *ama1*_*I-II*_ (0.108) *and msp1*_*42*_ (0.097), but a higher value for *dbp*_*II*_ (0.247). However, only in SMX were the three merozoite genes analyzed simultaneously (in the same parasite sample).

## Discussion

In Mexico the problem of malaria transmission has existed for a long time, and since the 1950’s the number of cases has been recorded. In the 80’s more than 100,000 cases were reported each year, at which time malaria was widespread in most of the country. From 2000 onwards, transmission has been gradually decreasing, now reduced to residual foci. Presumably, this is due to vector control, case detection and drug delivery [68]. In the last decade, Mexico has advanced to the pre-elimination phase [69]. The remaining *P. vivax* could correspond to parasites with genetic and biological characteristics that have allowed them to persistent in the affected regions. In the Americas, the scant information available on the diversity of the main merozoite vaccine candidates is fragmented e.g., *msp1*_*42*_ [11] *and dbp*_*II*_ [62] from BRZ, *ama1*_*I-II*_ from VEN [34], and *dbp*_*II*_ from COL [63].

**Fig. 4:**
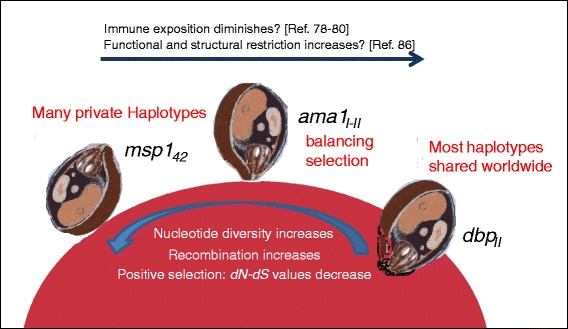
Summary of the genetic characteristics of *P. vivax* merozoite genes participating sequentially in erythrocyte invasion in southern Mexico. The parasite sample was obtained after hurricane Stan caused an increase in *P. vivax* transmission. MSP1_42_ participates in the initial adhesion to the reticulocyte, AMA1_I-II_ in re-orientation, and DBP_II_ in the moment that invasion initiates Ref. [7–9]

Figure 4 summarizes the differences between three merozoite genes of *P. vivax* in SMX. The present study evidences limited genetic diversity for *msp1*_*42*_ *> ama1*_*I-II*_ *> dbp*_*II*_. This low variation was previously detected for other makers such as *pvs25-28* [22] and the *circumsporozoite* gene [70]. Different biological and socioeconomic factors contribute to the maintenance of certain *P. vivax* diversity, including the rate of hypnozoite and asymptomatic blood infections, early gametocyte development, and the delay in diagnosis and treatment [71, 72]. These factors were probably magnified after hurricane Stan devastated the SMX region. Also, since 1999 in Mexico, malaria medication has been comprised of single doses of CQ and PQ in an intermittent monthly scheme. This medication scheme did not affect the hypnozoite prevalence per se [73, 74]. Therefore, most of the haplotypes detected in 2006 might correspond to the activation of genetically different hypnozoites in the region. After antimalarial measures reached these areas, fewer haplotypes were detected in 2007. Only those whose frequency was favored managed to prevail.

The haplotype networks arrangement of *msp1*_*42*_, *ama1*_*I-II*_*, dbp*_*II*_ suggested the presence of recombination and local diversification in these gene fragments in SMX. The greater complexity of the *msp1*_*42*_ network might be due to the high degree of recombination defined by the Rm value and linkage disequilibrium indexes. Multiple haplotype configurations were found in this hypo-endemic region, and probable aggravated in other locations with more complex epidemiology and higher transmission. Such finding evidenced the importance of evaluating the manner that multivalent vaccines components are evolving in the affected regions.

There is evidence that recombination events occur in *P. vivax* during meiotic and mitotic reproduction [32, 36]. Accordingly, it has been described that the mosaic structure of these merozoite genes resulted from recombination across the *dbp*_*II*_ [75], *msp1* [11] and *ama1*_*I-II*_ [34] gene sequences. Even in a low transmission setting, a high recombination rate for the *P. vivax msp1* gene occurs [11]. In SMX, the results suggest a moderate recombination rate for *msp1*_*42*_in the *P. vivax* sample. The presence of mixed genotype infections is a prerequisite for the generation of new recombinant haplotypes during meiosis in the mosquito midgut. In SMX, about 5–10 % of mixed infections were previously detected [23, 73]. Whether or not *msp1*_*42*_ recombinants were generated in SMX, they prevailed and local diversification probably occurred.

The nucleotide diversity found between SMX *P. vivax* merozoite genes and those at other locations could have been influenced by different historical, epidemiological and ecological transmission dynamics. For example, in SLK, with low and unstable transmission, recombination events in this gene fragment were reportedly numerous [13, 64, 76]. The chances of generating a recombinant were higher in SLK than SMX because 69 % of infections had more than one genotype [77].

These merozoite proteins are exposed to the host-specific immune response, which causes selective pressure likely results in an evasive parasite response [62, 78, 79]. At least in SMX, recombination events maintain nucleotide diversity in *msp1*_*42*_ as a function of immune evasion and likely counteract the low degree of positive selection. The latter is evidenced by the lower *dN-dS* value for *msp1*_*42*_ than for *ama1*_*I-II*_ and *dbp*_*II*_. Unlike in SMX, in most geographic locations the *dN-dS* value for *msp1*_*42*_ was highly significant.

Because of being processed on the merozoite surface, MSP1 is presumably more exposed to the immune system than AMA1 and DBP (Fig. 4). Studies in BRZ have reported that in response to natural infection, a higher proportion of individuals had detectable antibodies against MSP1 than AMA1: 95 vs 72.7 % [78] and 90.5 vs 57 % [80]. In addition, 58.3 % [79] and 65 % [18] of the antibody response was reported to be against domain II of AMA1 (AMA1_II_). On the other hand, DBP is only secreted at the time of invasion [15], and thus induces a weak and short-lasting antibody response [62], which in one study was detected in 44.5 % of primary infected individuals [78]. To evade the immune response, genes encoding for antigenic proteins accumulate non-synonymous mutations, which leads to an increase in diversity. In this study, values of *dN-dS* and Tajima’s D suggest that balancing selection [81] might have maintained the frequency of the *ama*_*I-II*_ haplotype in *P. vivax* from SMX, as has been suggested for other geographic sites such as SLK [13], IND [51], MYN [82], VNZ [34] and Honduras [83]. Patterns of diversifying selection were also suggested for *ama1*_*I-II*_ in VNZ [34] and COL [75].

Although Tajima’s D value was negative for *dbp*_*II*_ in SMX, no departure from neutrality was evidenced. Meanwhile, the *dN-dS* value (3.16) was highly significant and the absence of recombination may be indicative of a recent selective sweep. It has been reported that haplotype specific immunity existed for DBP_II_ [84]. Residues N417K, W437R and I503K were involved in antigenic drift; the resulting variant haplotypes KRK and NRK evaded antibodies induced by the Sal 1 sequence [14]. This type of polymorphisms are highly frequent at the global level [85]. In SMX, the haplotype bearing variant residues KRK was significantly more frequent (85 %). However, it is unknown whether its transmission was actually favored by other polymorphisms or by other genetic and/or biological factors. On the other hand, because *dbp*_*II*_ had the lowest nucleotide diversity, there were a large number of globally shared, closely related haplotypes. Hence, this gene fragment may be under functional and/or structural restrictions, as previously suggested by others [86] (Fig. 4).

The F_ST_ values of the current study indicate that all merozoite gene fragments evidence a certain degree of *P. vivax* genetic differentiation in SMX. Consistently higher values were obtained for *dbp*_*II*_, which showed almost no recombination and no significant departure from neutrality, resulting in little diversity. However, if antigenic drift was operating in this gene fragment, it may have rendered few divergent *dbp*_*II*_ haplotypes and biased the F_ST_ values. A similar effect was observed for *msp1*_*42*_ in TUR parasites [43]. Notwithstanding, in order to elucidate *P. vivax* differentiation and the degree of gene flow in SMX, it would be necessary to analyze mitochondrial markers. The existence of genetic flow between Central and South American regions, which has been suggested [87], is an important factor to be taken into account for malaria control and elimination.

## Conclusions

The aforementioned results of the present study provide insights into merozoite vaccine candidates in Mesoamerica. Several factors should be considered to design and monitor monovalent and multivalent formulates, including the fluctuation of haplotype frequency, differences in intensity of recombination and natural selection of vaccine components [88–90]. In addition, the current findings can also facilitate the establishment of a molecular baseline for epidemiological surveillance.

## Additional files

Additional file 1:
***P. vivax*** ***dbp***_***II***_ **polymorphism in isolates from southern Mexico, 2006–2007.** (XLSX 12 kb) Additional file 2:
***P. vivax ama1***_***I-II***_ **polymorphisms in isolates from southern Mexico, 2006–2007.** (XLSX 12 kb) Additional file 3:
***P. vivax msp1***_***42***_ **polymorphisms in isolates from southern Mexico, 2006–2007.** (XLSX 13 kb) Additional file 4:
***P. vivax dbp***_***II***_ **,** ***ama1***_***I-II***_ **and** ***msp1***_***42***_ **; haplotypes detected in southern Mexico, 2006–2007.** (DOCX 41 kb) Additional file 5:
**Comparison of the genetic diversity of** ***P. vivax*** **merozoite proteins between southern Mexico and other geographic sites.** (DOCX 48 kb) Additional file 6:
***P. vivax*** **merozoite genes: shared haplotypes between southern Mexico and other geographic sites.** (DOCX 39 kb) Additional file 7:
**Natural selection and minimal number of recombination events for** ***P. vivax*** **merozoite proteins from different geographic origins.** (DOCX 47 kb)

## Abbreviations

*P. vivax*: *Plasmodium vivax*
DBP: duffy binding protein
MSP1: merozoite surface protein 1
AMA1: apical membrane antigen 1
*dbp*_*II*_: domain II of the duffy binding protein gene
*ama1*_*I-II*_: domain I and II of the apical membrane antigen 1 gene
*msp1*_*42*_: carboxyl fragment of 42 kD of the merozoite surface protein 1 gene
Rm: minimal number of recombination events
SMX: southern Mexico
TUR: Turkey
BRZ: Brazil
SLK: Sri Lanka
THL: Thailand
MYN: Myanmar
VEN: Venezuela
COL: Colombia
IRN: Iran
IND: India
PNG: Papua New Guinea
SK: South Korea
SNG: Singapore
BNG: Bangladesh
CAM: Cambodia

## References

[1] Guerra CA, Howes RE, Patil AP, Gething PW, Van Boeckel TP, Temperley WH (2010). The international limits and population at risk of Plasmodium vivax transmission in 2009. PLoS Negl Trop Dis.

[2] WHO. World Malaria Report 2012. World Health Organization, Geneva. http://www.who.int/malaria/publications/world_malaria_report_2012/en/. Accessed 17 Dec 2015.

[3] WHO. World Malaria Report 2013. World Health Organization, Geneva. http://www.who.int/malaria/publications/world_malaria_report_2013/en/. Accessed 17 Dec 2015.

[4] PAHO. Interactive Malaria Statistics 2012. Pan american Health Organization, Washington. http://www.paho.org/hq/index.php?option=com_content&view=article&id=2632%3A2010-interactive-malaria-statistics&catid=1617%3Amalaria-statistics-maps&Itemid=2130&lang=es. Accessed 17 Dec 2015.

[5] Carter KH, Singh P, Mujica OJ, Escalada RP, Ade MP, Castellanos LG (2015). Malaria in the Americas: Trends from 1959 to 2011. Am J Trop Med Hyg.

[6] WHO. World Malaria Report 2014. World Health Organization, Geneva. http://www.who.int/malaria/publications/world_malaria_report_2014/en/. Accessed 17 Dec 2015.

[7] Holder AA, Guevara Patiño JA, Uthaipibull C, Syed SE, Ling IT, Scott-Finnigan T (1999). Merozoite surface protein 1, immune evasion, and vaccines against asexual blood stage malaria. Parasitologia.

[8] Pizarro JC, Normand BV-L, Chesne-Seck M-L, Collins CR, Withers-Martinez C, Hackett F (2005). Crystal Structure of the Malaria Vaccine Candidate Apical Membrane Antigen 1. Science.

[9] Iyer J, Grüner AC, Rénia L, Snounou G, Preiser PR (2007). Invasion of host cells by malaria parasites: a tale of two protein families. Mol Microbiol.

[10] Tsuboi T, Kappe SH, Al-Yaman F, Prickett MD, Alpers M, Adams JH (1994). Natural variation within the principal adhesion domain of the Plasmodium vivax duffy binding protein. Infect Immun.

[11] Putaporntip C, Jongwutiwes S, Sakihama N, Ferreira MU, Kho W-G, Kaneko A (2002). Mosaic organization and heterogeneity in frequency of allelic recombination of the Plasmodium vivax merozoite surface protein-1 locus. Proc Natl Acad Sci U S A.

[12] Bonilla JA, Validum L, Cummings R, Palmer CJ (2006). Genetic diversity of Plasmodium vivax Pvcsp and Pvmsp1 in Guyana, South America. Am J Trop Med Hyg.

[13] Gunasekera AM, Wickramarachchi T, Neafsey DE, Ganguli I, Perera L, Premaratne PH (2007). Genetic diversity and selection at the Plasmodium vivax apical membrane antigen-1 (PvAMA-1) locus in a Sri Lankan population. Mol Biol Evol.

[14] VanBuskirk KM, Cole-Tobian JL, Baisor M, Sevova ES, Bockarie M, King CL (2004). Antigenic drift in the ligand domain of Plasmodium vivax duffy binding protein confers resistance to inhibitory antibodies. J Infect Dis.

[15] Singh SK, Hora R, Belrhali H, Chitnis CE, Sharma A (2006). Structural basis for Duffy recognition by the malaria parasite Duffy-binding-like domain. Nature.

[16] Bastos MS, da Silva-Nunes M, Malafronte RS, Hoffmann EH, Wunderlich G, Moraes SL (2007). Antigenic polymorphism and naturally acquired antibodies to Plasmodium vivax merozoite surface protein 1 in rural Amazonians. Clin Vaccine Immunol.

[17] Zeyrek FY, Babaoglu A, Demirel S, Erdogan DD, Ak M, Korkmaz M (2008). Analysis of naturally acquired antibody responses to the 19-kd C-terminal region of merozoite surface protein-1 of Plasmodium vivax from individuals in Sanliurfa, Turkey. Am J Trop Med Hyg.

[18] Múfalo BC, Gentil F, Bargieri DY, Costa FTM, Rodrigues MM, Soares IS (2008). Plasmodium vivax apical membrane antigen-1: comparative recognition of different domains by antibodies induced during natural human infection. Microb Infect.

[19] Vicentin EC, Françoso KS, Rocha MV, Iourtov D, dos Santos FL, Kubrusly FS (2014). Invasion-Inhibitory Antibodies Elicited by Immunization with Plasmodium vivax Apical Membrane Antigen-1 Expressed in Pichia pastoris Yeast. Infect Immun.

[20] Grimberg BT, Udomsangpetch R, Xainli J, McHenry A, Panichakul T, Sattabongkot J (2007). Plasmodium vivax Invasion of Human Erythrocytes Inhibited by Antibodies Directed against the Duffy Binding Protein. PLoS Med.

[21] Kim S-H, Hwang S-Y, Lee Y-S, Choi I-H, Park S-G, Kho W-G (2007). Single-Chain Antibody Fragment Specific for Plasmodium vivax Duffy Binding Protein. Clin Vaccine Immunol.

[22] Gonzalez-Ceron L, Alvarado-Delgado A, Martinez-Barnetche J, Rodriguez MH, Ovilla-Munoz M, Perez F (2010). Sequence variation of ookinete surface proteins Pvs25 and Pvs28 of Plasmodium vivax isolates from Southern Mexico and their association to local anophelines infectivity. Infect Genet Evol.

[23] Joy DA, Gonzalez-Ceron L, Carlton JM, Gueye A, Fay M, McCutchan TF (2008). Local adaptation and vector-mediated population structure in Plasmodium vivax malaria. Mol Biol Evol.

[24] Cerritos R, González-Cerón L, Nettel JA, Wegier A (2014). Genetic structure of Plasmodium vivax using the merozoite surface protein 1 icb5-6 fragment reveals new hybrid haplotypes in southern Mexico. Malar J.

[25] Boletín Epidemiológico, Ministry of Health. Mexico. 2005. http://www.epidemiologia.salud.gob.mx/dgae/boletin/intd_boletin.html. Accessed 17 Dec 2015.

[26] Librado P, Rozas J (2009). DnaSP v5: a software for comprehensive analysis of DNA polymorphism data. Bioinformatics.

[27] Clement M, Posada D, Crandall KA (2000). TCS: a computer program to estimate gene genealogies. Mol Ecol.

[28] Bandelt HJ, Forster P, Röhl A (1999). Median-joining networks for inferring intraspecific phylogenies. Mol Biol Evol.

[29] Hudson RR, Kaplan NL (1985). Statistical Properties of the Number of Recombination Events in the History of a Sample of DNA Sequences. Genetics.

[30] Escalante AA, Cornejo OE, Rojas A, Udhayakumar V, Lal AA (2004). Assessing the effect of natural selection in malaria parasites. Trends Parasitol.

[31] Thakur A, Alam MT, Sharma YD (2008). Genetic diversity in the C-terminal 42 kDa region of merozoite surface protein-1 of Plasmodium vivax (PvMSP-1(42)) among Indian isolates. Acta Trop.

[32] Dias S, Longacre S, Escalante AA, Udagama-Randeniya PV (2011). Genetic diversity and recombination at the C-terminal fragment of the merozoite surface protein-1 of Plasmodium vivax (PvMSP-1) in Sri Lanka. Infect Genet Evol.

[33] Pacheco MA, Poe AC, Collins WE, Lal AA, Tanabe K, Kariuki SK (2007). A comparative study of the genetic diversity of the 42 kDa fragment of the merozoite surface protein 1 in Plasmodium falciparum and P. vivax. Infect Genet Evol.

[34] Ord RL, Tami A, Sutherland CJ (2008). ama1 genes of sympatric Plasmodium vivax and P. falciparum from Venezuela differ significantly in genetic diversity and recombination frequency. PLoS One.

[35] Gosi P, Khusmith S, Khalambaheti T, Lanar DE, Schaecher KE, Fukuda MM (2008). Polymorphism patterns in Duffy-binding protein among Thai Plasmodium vivax isolates. Malar J.

[36] Cole-Tobian J, King CL (2003). Diversity and natural selection in Plasmodium vivax Duffy binding protein gene. Mol Biochem Parasitol.

[37] Nei M, Gojobori T (1986). Simple methods for estimating the numbers of synonymous and nonsynonymous nucleotide substitutions. Mol Biol Evol.

[38] Tamura K, Stecher G, Peterson D, Filipski A, Kumar S (2013). MEGA6: Molecular Evolutionary Genetics Analysis Version 6.0. Mol Biol Evol.

[39] Wright S (1951). The genetical structure of populations. Ann Eugenics.

[40] Rozas J, Sanchez-del Barrio JC, Messeguer X, Rozas R (2003). DnaSP, DNA polymorphism analyses by the coalescent and other methods. Bioinformatics.

[41] Kang J-M, Ju H-L, Kang Y-M, Lee D-H, Moon S-U, Sohn W-M (2012). Genetic polymorphism and natural selection in the C-terminal 42 kDa region of merozoite surface protein-1 among *Plasmodium vivax* Korean isolates. Malar J.

[42] Han E-T, Wang Y, Lim CS, Cho JH, Chai J-Y (2011). Genetic diversity of the malaria vacine candidate merozoite surface protein 1 gene of Plasmodium vivax field isolates in Republic of Korea. Parasitol Res.

[43] Zeyrek FY, Tachibana S-I, Yuksel F, Doni N, Palacpac N, Arisue N (2010). Limited Polymorphism of the Plasmodium vivax Merozoite Surface Protein 1 Gene in Isolates from Turkey. Am J Trop Med Hyg.

[44] Jongwutiwes S, Putaporntip C, Hughes AL (2010). Bottleneck Effects on Vaccine-Candidate Antigen Diversity of Malaria Parasites in Thailand. Vaccine.

[45] Putaporntip C, Jongwutiwes S, Seethamchai S, Kanbara H, Tanabe K (2000). Intragenic recombination in the 3′ portion of the merozoite surface protein 1 gene of *Plasmodium vivax*. Mol Biochem Parasitol.

[46] Ng LC, Lee KS, Tan CH, Ooi PL, Lam-Phua SG, Lin R (2010). Entomologic and molecular investigation into Plasmodium vivax transmission in Singapore, 2009. Malar J.

[47] Sheikh IH, Kaushal DC, Singh V, Kumar N, Chandra D, Kaushal NA (2014). Cloning, overexpression and characterization of soluble 42 kDa fragment of merozoite surface protein-1 of Plasmodium vivax. Protein Expr Purif.

[48] Parobek CM, Bailey JA, Hathaway NJ, Socheat D, Rogers WO, Juliano JJ (2014). Differing Patterns of Selection and Geospatial Genetic Diversity within Two Leading Plasmodium vivax Candidate Vaccine Antigens. PLoS Negl Trop Dis.

[49] Putaporntip C, Jongwutiwes S, Grynberg P, Cui L, Hughes AL (2009). Nucleotide sequence polymorphism at the apical membrane antigen-1 locus reveals population history of Plasmodium vivax in Thailand. Infect Genet Evol.

[50] Rajesh V, Elamaran M, Vidya S, Gowrishankar M, Kochar D, Das A (2007). Plasmodium vivax: genetic diversity of the apical membrane antigen-1 (AMA-1) in isolates from India. Exp Parasitol.

[51] Thakur A, Alam MT, Bora H, Kaur P, Sharma YD (2008). Plasmodium vivax: sequence polymorphism and effect of natural selection at apical membrane antigen 1 (PvAMA1) among Indian population. Gene.

[52] Zakeri S, Sadeghi H, Mehrizi AA, Djadid ND (2013). Population genetic structure and polymorphism analysis of gene encoding apical membrane antigen-1 (AMA-1) of Iranian Plasmodium vivax wild isolates. Acta Trop.

[53] Esmaelli Rastaghi AR, Nedaei F, Navhrevanian H, Hoseinkhan N (2014). Genetic diversity and effect of natural selection at apical membrane antigen-1 (AMA-1) among Iranian Plasmodium vivax isolates. Folia Parasitol (Praha).

[54] Arnott A, Mueller I, Ramsland PA, Siba PM, Reeder JC, Barry AE (2013). Global Population Structure of the Genes Encoding the Malaria Vaccine Candidate, Plasmodium vivax Apical Membrane Antigen 1 (PvAMA1). PLoS Negl Trop Dis.

[55] Ju H-L, Kang J-M, Moon S-U, Kim J-Y, Lee H-W, Lin K (2012). Genetic polymorphism and natural selection of Duffy binding protein of Plasmodium vivax Myanmar isolates. Malar J.

[56] Ju HL, Kang JM, Moon SU, Bahk YY, Cho PY, Sohn WM (2013). Genetic diversity and natural selection of Duffy binding protein of Plasmodium vivax Korean isolates. Acta Trop.

[57] Kho W-G, Chung J-Y, Sim E-J, Kim D-W, Chung W-C (2001). Analysis of polymorphic regions of Plasmodium vivax Duffy binding protein of Korean isolates. Korean J Parasitol.

[58] Suh IB, Hoffman KJ, Kim SH, Song KJ, Song JW, Lee JS (2001). The analysis of Plasmodium vivax Duffy receptor binding domain gene sequence from resurgent Korea isolates. Parasitol Res.

[59] Xainli J, Adams JH, King CL (2000). The erythrocyte binding motif of plasmodium vivax duffy binding protein is highly polymorphic and functionally conserved in isolates from Papua New Guinea. Mol Biochem Parasitol.

[60] Cole-Tobian JL, Cortés A, Baisor M, Kastens W, Xainli J, Bockarie M (2002). Age-Acquired Immunity to a Plasmodium vivax Invasion Ligand, the Duffy Binding Protein. J Infect Dis.

[61] Cole-Tobian JL, Biasor M, King CL (2005). High complexity of Plasmodium vivax infections in Papua New Guinean children. Am J Trop Med Hyg.

[62] Sousa TN, Tarazona-Santos EM, Wilson DJ, Madureira AP, Falcão PRK, Fontes CJF (2010). Genetic variability and natural selection at the ligand domain of the Duffy binding protein in brazilian Plasmodium vivax populations. Malar J.

[63] Ampudia E, Patarroyo MA, Patarroyo ME, Murillo LA (1996). Genetic polymorphism of the Duffy receptor binding domain of Plasmodium vivax in Colombian wild isolates. Mol Biochem Parasitol.

[64] Premaratne PH, Aravinda BR, Escalante AA, Udagama PV (2011). Genetic diversity of Plasmodium vivax Duffy Binding Protein II (PvDBPII) under unstable transmission and low intensity malaria in Sri Lanka. Infect Genet Evol.

[65] Babaeekho L, Zakeri S, Djadid ND (2009). Genetic mapping of the duffy binding protein (DBP) ligand domain of Plasmodium vivax from unstable malaria region in the Middle East. Am J Trop Med Hyg.

[66] Valizadeh V, Zakeri S, Mehrizi AA, Djadid ND (2014). Population genetics and natural selection in the gene encoding the Duffy binding protein II in Iranian Plasmodium vivax wild isolates. Infect Genet Evol.

[67] Tetteh KKA, Stewart LB, Ochola LI, Amambua-Ngwa A, Thomas AW, Marsh K (2009). Prospective Identification of Malaria Parasite Genes under Balancing Selection. PLoS One.

[68] Norma Oficial Mexicana para la vigilancia epidemiológica, prevención y diagnóstico de enfermedades transmitidas por vector (NOM-032-SSA2-2002). Ministry of Health, Mexico. 2002. http://www.salud.gob.mx/unidades/cdi/nom/032ssa202.html. Accessed 17 Dec 2015.

[69] WHO. World Malaria Report 2009. World Health Organization, Geneva. http://www.who.int/malaria/world_malaria_report_2009/en/. Accessed 17 Dec 2015.

[70] Gonzalez-Ceron L, Martinez-Barnetche J, Montero-Solis C, Santillan F, Soto AM, Rodriguez MH (2013). Molecular epidemiology of Plasmodium vivax in Latin America: polymorphism and evolutionary relationships of the circumsporozoite gene. Malar J.

[71] Barry AE, Waltmann A, Koepfli C, Barnadas C, Mueller I (2015). Uncovering the transmission dynamics of Plasmodium vivax using population genetics. Pathog Glob Health.

[72] Mendis K, Sina BJ, Marchesini P, Carter R (2001). The neglected burden of Plasmodium vivax malaria. Am J Trop Med Hyg.

[73] Gonzalez-Ceron L, Mu J, Santillan F, Joy D, Sandoval MA, Camas G (2013). Molecular and epidemiological characterization of Plasmodium vivax recurrent infections in southern Mexico. Parasit Vectors.

[74] Gonzalez-Ceron L, Rodriguez MH, Sandoval MA, Santillan F, Galindo-Virgen S, Betanzos AF (2015). Effectiveness of combined chloroquine and primaquine treatment in 14 days versus intermittent single dose regimen, in an open, non-randomized, clinical trial, to eliminate Plasmodium vivax in southern Mexico. Malar J.

[75] Martinez P, Suarez CF, Cardenas PP, Patarroyo MA (2004). Plasmodium vivax Duffy binding protein: a modular evolutionary proposal. Parasitology.

[76] Dias S, Somarathna M, Manamperi A, Escalante AA, Gunasekera AM, Udagama PV (2011). Evaluation of the genetic diversity of domain II of Plasmodium vivax Apical Membrane Antigen 1 (PvAMA-1) and the ensuing strain-specific immune responses in patients from Sri Lanka. Vaccine.

[77] Gunawardena S, Ferreira MU, Kapilananda GMG, Wirth DF, Karunaweera ND (2014). The Sri Lankan paradox: high genetic diversity in Plasmodium vivax populations despite decreasing levels of malaria transmission. Parasitology.

[78] Barbedo MB, Ricci R, Jimenez MCS, Cunha MG, Yazdani SS, Chitnis CE (2007). Comparative recognition by human IgG antibodies of recombinant proteins representing three asexual erythrocytic stage vaccine candidates of Plasmodium vivax. Mem Inst Oswaldo Cruz.

[79] Bueno LL, Lobo FP, Morais CG, Mourão LC, de Ávila RAM, Soares IS (2011). Identification of a Highly Antigenic Linear B Cell Epitope within Plasmodium vivax Apical Membrane Antigen 1 (AMA-1). PLoS One.

[80] Oliveira TR, Fernandez-Becerra C, Jimenez MCS, Del Portillo HA, Soares IS (2006). Evaluation of the acquired immune responses to Plasmodium vivax VIR variant antigens in individuals living in malaria-endemic areas of Brazil. Malar J.

[81] Arnott A, Barnadas C, Senn N, Siba P, Mueller I, Reeder JC (2013). High genetic diversity of Plasmodium vivax on the north coast of Papua New Guinea. Am J Trop Med Hyg.

[82] Moon S-U, Na B-K, Kang J-M, Kim J-Y, Cho S-H, Park Y-K (2010). Genetic polymorphism and effect of natural selection at domain I of apical membrane antigen-1 (AMA-1) in Plasmodium vivax isolates from Myanmar. Acta Trop.

[83] Lopez AC, Ortiz A, Coello J, Sosa-Ochoa W, Torres REM, Banegas EI (2012). Genetic diversity of Plasmodium vivax and Plasmodium falciparum in Honduras. Malar J.

[84] Cole-Tobian JL, Michon P, Biasor M, Richards JS, Beeson JG, Mueller I (2009). Strain-Specific Duffy Binding Protein Antibodies Correlate with Protection against Infection with Homologous Compared to Heterologous Plasmodium vivax Strains in Papua New Guinean Children. Infect Immun.

[85] Nóbrega de Sousa T, Carvalho LH, de Brito CF A (2011). Worldwide Genetic Variability of the Duffy Binding Protein: Insights into Plasmodium vivax Vaccine Development. PLoS One.

[86] Putaporntip C, Jongwutiwes S, Tanabe K, Thaithong S (1997). Interallelic recombination in the merozoite surface protein 1 (MSP-1) gene of Plasmodium vivax from Thai isolates. Mol Biochem Parasitol.

[87] Taylor JE, Pacheco MA, Bacon DJ, Beg MA, Dantas Machado RL, Fairhurst RM (2013). The evolutionary history of Plasmodium vivax as inferred from mitochondrial genomes: parasite genetic diversity in the Americas. Mol Biol Evol.

[88] Terheggen U, Drew DR, Hodder AN, Cross NJ, Mugyenyi CK, Barry AE (2014). Limited antigenic diversity of Plasmodium falciparum apical membrane antigen 1 supports the development of effective multi-allele vaccines. BMC Med.

[89] Tran TM, Portugal S, Draper SJ, Crompton PD (2015). Malaria Vaccines: Moving Forward After Encouraging First Steps. Curr Trop Med Rep.

[90] Spiegel H, Boes A, Kastilan R, Kapelski S, Edgue G, Beiss V (2015). The stage-specific in vitro efficacy of a malaria antigen cocktail provides valuable insights into the development of effective multi-stage vaccines. Biotech J.

