# Supplementary material for: Diversity and evolutionary genetics of the three major Plasmodium vivax merozoite genes participating in reticulocyte invasion in southern Mexico
Source: Parasit Vectors. 2015 Dec 21;8:651. doi: 10.1186/s13071-015-1266-7 (PMC4687067; doi:10.1186/s13071-015-1266-7)
Supplement: Additional file 4: — P. vivax dbp II , ama1 I-II and msp1 42 ; haplotypes detected in southern Mexico, 2006–2007. (DOCX 41 kb) [file 13071_2015_1266_MOESM4_ESM.docx]

**Additional file 4** *P. vivax* *dbp_II_*, *ama1_I-II_* and *msp1_42_*; haplotypes detected in southern Mexico, 2006-2007.

| **Date of collection** | **Sample code** | **Haplotypes:** | | | |
| --- | --- | --- | --- | --- | --- |
|  |  | ***dbp_II_*** | ***ama1_I-II_*** | ***msp1_42_*** | **Combined haplotype** |
| 10/01/2006 | GA1 | dh2 | ah2 | mh1 | F |
| 19/01/2006 | MG | dh4 | ah1 | mh2 | L |
| 14/02/2006 | TO | dh2 | ah1 | mh2 | E |
| 11/03/2006 | GA2 | dh1 | ah3 | mh2 | C |
| 23/03/2006 | TA1 | dh1 | ah6 | mh1 | J |
| 06/04/2006 | SR | dh1 | ah3 | mh2 | C |
| 12/04/2006 | CE | dh1 | ah1 | mh6 | I |
| 04/05/2006 | FB | dh1 | ah2 | mh3 | B |
| 27/05/2006 | TA2 | dh5 | ah2 | mh8 | M |
| 01/06/2006 | VI1 | dh1 | ah2 | mh3 | B |
| 14/06/2006 | TA3 | dh1 | ah2 | mh3 | B |
| 26/08/2006 | HU | dh8 | ah7 | mh7 | P |
| 11/09/2006 | CN | dh3 | ah4 | mh4 | G |
| 28/10/2006 | CB | dh7 | ah4 | mh4 | O |
| 15/11/2006 | TA4 | dh6 | ah5 | mh9 | N |
| 21/11/2006 | UR | dh2 | ah1 | mh2 | E |
| 17/12/2006 | ZA | dh2 | ah3 | mh2 | K |
| 06/01/2007 | GA3 | dh1 | ah1 | mh1 | A |
| 11/01/2007 | CA | dh1 | ah3 | mh2 | C |
| 12/01/2007 | MR | dh1 | ah2 | mh5 | D |
| 21/01/2007 | VI2 | dh1 | ah1 | mh1 | A |
| 27/02/2007 | RE1 | dh1 | ah1 | mh1 | A |
| 26/03/2007 | RE2 | dh1 | ah1 | mh1 | A |
| 26/04/2007 | CH | dh3 | ah4 | mh4 | G |
| 07/05/2007 | GU | dh1 | ah1 | mh1 | A |
| 22/05/2007 | LA | dh1 | ah1 | mh2 | H |
| 30/05/2007 | PR | dh1 | ah2 | mh3 | B |
| 01/06/2007 | TA5 | dh1 | ah1 | mh1 | A |
| 10/06/2007 | NV | dh1 | ah2 | mh5 | D |
| 28/06/2007 | TA6 | dh1 | ah1 | mh1 | A |
| 03/07/2007 | TA7 | dh1 | ah1 | mh1 | A |
| 07/07/2007 | TA8 | dh1 | ah1 | mh1 | A |
| 15/09/2007 | TA9 | dh1 | ah1 | mh1 | A |
| 21/09/2007 | TA10 | dh1 | ah1 | mh1 | A |
| 06/11/2007 | TL | dh2 | ah2 | mh1 | F |
