# Supplementary material for: Diversity and evolutionary genetics of the three major Plasmodium vivax merozoite genes participating in reticulocyte invasion in southern Mexico
Source: Parasit Vectors. 2015 Dec 21;8:651. doi: 10.1186/s13071-015-1266-7 (PMC4687067; doi:10.1186/s13071-015-1266-7)
Supplement: Additional file 5: — Comparison of the genetic diversity of P. vivax merozoite proteins between southern Mexico and other geographic sites. (DOCX 48 kb) [file 13071_2015_1266_MOESM5_ESM.docx]

**Additional file 5** Comparison of the genetic diversity of *P. vivax* merozoite proteins between Southern Mexico and other geographic sites.

In order to add as many gene sequences as possible, the length of the nucleotide sequences was edited. *Msp1_42_* sequences were trimmed to 981 pb (nucleotides 4,150-5131, resulting in the omission of three nucleotide changes, two synonymous (at codons 1356 and 1357) and one non-synonymous change (at codon 1361). Similarly, *dbp_II_* sequences were trimmed to a fragment of 663 pb (881 – 1543 nt), leaving out four nucleotide changes, one synonymous (codon 530) and three non-synonymous (codons 220, 529 and 551). *Ama1_I-II_* sequences were trimmed to 780 pb (376 – 1155 nt), but no polymorphic site of the SMX group was excluded.

| **a. *msp1_42_*** | N | NT | SS | H | Hd (SD) | | **π (SD)** | | Θ-w ± (SD nr, fr) | |
| --- | --- | --- | --- | --- | --- | --- | --- | --- | --- | --- |
| TUR | 30 | 27 | 27 | 3 | 0.536 | (0.077) | 0.00735 | (0.00158) | 0.00695 | (0.00247, 0.00134) |
| SK | 200 | 50 | 47 | 10 | 0.871 | (0.006) | 0.01633 | (0.00064) | 0.00816 | (0.00209, 0.00119) |
| **SMX** | **35** | **56** | **49** | **8** | **0.778** | **(0.050)** | **0.01791** | **(0.00195)** | **0.01213** | **(0.00394, 0.00173)** |
| SNG | 50 | 97 | 89 | 27 | 0.898 | (0.031) | 0.01914 | (0.00144) | 0.02025 | (0.00592, 0.00215) |
| THA | 93 | 62 | 55 | 43 | 0.965 | (0.009) | 0.02259 | (0.0006) | 0.01098 | (0.00303, 0.00148) |
| BRZ | 11 | 58 | 53 | 8 | 0.945 | (0.054) | 0.02287 | (0.00309) | 0.01845 | (0.00758, 0.00253) |
| CAM | 44 | 63 | 57 | 26 | 0.950 | (0.018) | 0.02312 | (0.00061) | 0.01336 | (0.00412, 0.00177) |
| MYN | 28 | 64 | 59 | 26 | 0.995 | (0.011) | 0.02403 | (0.00104) | 0.01546 | (0.00516, 0.00201) |
| SLK | 106 | 70 | 63 | 47 | 0.969 | (0.006) | 0.02425 | (0.00045) | 0.01227 | (0.00327, 0.00155) |
| IND-BN | 35 | 69 | 61 | 30 | 0.988 | (0.011) | 0.02431 | (0.00064) | 0.0151 | (0.00482, 0.00193) |
| Global | 632 | 133 | 117 | 185 | 0.9737 | (0.0026) | 0.02379 | (0.00017) | 0.01698 | (0.00342, 0.00157) |
| **b. *ama1_I-II_*** | N | NT | SS | H | Hd (SD) | | **π (SD)** | | Θ-w ± (SD nr, fr) | |
| VNZ | 73 | 16 | 16 | 17 | 0.907 | (0.015) | 0.00682 | (0.00024) | 0.00422 | (0.00148, 0.00106) |
| **SMX** | **35** | **14** | **14** | **7** | **0.723** | **(0.057)** | **0.00846** | **(0.00045)** | **0.00436** | **(0.00170, 0.00116)** |
| PNG | 102 | 32 | 29 | 66 | 0.989 | (0.003) | 0.00908 | (0.00035) | 0.00715 | (0.00214, 0.00133) |
| SLK | 23 | 26 | 25 | 15 | 0.949 | (0.028) | 0.01017 | (0.00092) | 0.00868 | (0.00326, 0.00174) |
| THL | 231 | 41 | 37 | 89 | 0.927 | (0.012) | 0.01052 | (0.0003) | 0.00788 | (0.00207, 0.00130) |
| *IND | 60 | 36 | 34 | 51 | 0.993 | (0.005) | 0.01081 | (0.00035) | 0.00935 | (0.00291, 0.00160) |
| IRN | 83 | 38 | 35 | 55 | 0.985 | (0.005) | 0.01097 | (0.00034) | 0.00899 | (0.00267, 0.00152) |
| Global | 607 | 67 | 60 | 273 | 0.9856 | (0.002) | 0.01107 | (0.00015) | 0.01101 | (0.00243, 0.00142) |
| **c. *dbp_II_*** | N | NT | SS | H | Hd (SD) | | **π (SD)** | | Θ-w ± (SD nr, fr) | |
| **SMX** | **35** | **10** | **10** | **7** | **0.553** | **(0.092)** | **0.00406** | **(0.00078)** | **0.00366** | **(0.00155, 0.00116)** |
| SK | 111 | 67 | 62 | 21 | 0.778 | (0.028) | 0.00570 | (0.00088) | 0.01770 | (0.00470, 0.00225) |
| IRN | 130 | 19 | 19 | 14 | 0.794 | (0.022) | 0.00659 | (0.00027) | 0.00527 | (0.00168, 0.00121) |
| IND | 95 | 38 | 36 | 33 | 0.912 | (0.019) | 0.00814 | (0.00048) | 0.01059 | (0.00308, 0.00177) |
| BRZ | 122 | 20 | 20 | 34 | 0.934 | (0.012) | 0.00828 | (0.00034) | 0.00561 | 0.00178, 0.00125) |
| COL | 18 | 16 | 16 | 17 | 0.993 | (0.021) | 0.0091 | (0.00069) | 0.00702 | (0.00292, 0.00175) |
| PNG | 201 | 72 | 71 | 71 | 0.914 | (0.012) | 0.00977 | (0.00033) | 0.01822 | (0.00442, 0.00216) |
| SLK | 100 | 27 | 27 | 39 | 0.922 | (0.014) | 0.00986 | (0.00053) | 0.00787 | (0.00239, 0.00151) |
| MYN | 54 | 27 | 27 | 12 | 0.875 | (0.029) | 0.01025 | (0.00060) | 0.00894 | (0.00292, 0.00172) |
| THL | 30 | 39 | 38 | 24 | 0.982 | (0.014) | 0.01175 | (0.00098) | 0.01449 | (0.00496, 0.00235) |
| Global | 896 | 192 | 173 | 214 | 0.967 | (0.002) | 0.00943 | (0.00019) | 0.03544 | 0.00662, 0.00269) |

***^a^***981bp, ***^b^***780bp and ***^c^***663bp

SMX, Southern Mexico; IRN, Iran; IND, India; BRZ, Brazil; COL, Colombia; PNG, Papua New Guinea; SLK, Sri Lanka; THL, Thailand; SK, South Korea; MYN, Myanmar; VNZ, Venezuela; SNG, Singapore; BNG, Bangladesh; TUR, Turkey

N, number of isolates; NT, number of mutations, SS, segregating sites; Hd, haplotype diversity; π, nucleotide diversity; θ, genetic diversity; SD, standard deviation: nr, no recombining and fr, free recombination.

Only in SMX the three gene fragments were analyzed in the same parasite sample, results from other sites were for different parasite samples
