# Supplementary material for: Diversity and evolutionary genetics of the three major Plasmodium vivax merozoite genes participating in reticulocyte invasion in southern Mexico
Source: Parasit Vectors. 2015 Dec 21;8:651. doi: 10.1186/s13071-015-1266-7 (PMC4687067; doi:10.1186/s13071-015-1266-7)
Supplement: Additional file 6: — P. vivax merozoite genes: shared haplotypes between southern Mexico and other geographic sites. (DOCX 39 kb) [file 13071_2015_1266_MOESM6_ESM.docx]

**Additional file 6** Comparison of the haplotype detection and its frequency of *P. vivax* between southern Mexico and other geographic sites

| ***^a^dbp_II_***  **SMX (%)** | **Shared haplotypes**  **(%)** | ***^b^ama1_I-II_***  **SMX (%)** | **Shared haplotypes (%)** | ***^c^msp1_42_***  **SMX (%)** | **Shared haplotypes (%)** |
| --- | --- | --- | --- | --- | --- |
| dh1(65.7) | SK(11.7),THL (3.3), BRZ(5.7), IRN(17.7) | ah1(45.7) | - | mh1(40) | SK(16.5) TUR(63.3), BRZ(18.2) |
| dh2(14.3) | BRZ(1.6), IND(1), SLK(1) | ah2(25.7) | - | mh2(22.8) | - |
| dh3-h7(8.5) | THL(3.3), SLK(1), BRZ(2.4), IND(9.5) | ah3(11.4) | VNZ (6.3) | mh3(11.4) | THL(4.3) |
| dh4(2.8) | BRZ(2.4), IRN(1.5), IND (4.2) | ah4(8.5) | VNZ (5.2) | mh4/mh7*(11.3) | - |
| dh5(2.8) | - | ah5(2.8) | - | mh5(5.7) | - |
| dh6(2.8) | - | ah6(2.8) | - | mh6(2.8) | - |
| dh8(2.8) | BRZ(18.5), IND(24.2), SLK(18) | ah7(2.8) | - | mh8(2.8) | - |
|  |  |  |  | mh9(2.8) | - |

***^a^***981bp, ***^b^***780bp and ***^c^***663bp

SMX, Southern Mexico; IRN, Iran; IND, India; BRZ, Brazil; SLK, Sri Lanka; THL, Thailand; SK, South Korea; VNZ, Venezuela; TUR, Turkey.
