# Supplementary material for: Diversity and evolutionary genetics of the three major Plasmodium vivax merozoite genes participating in reticulocyte invasion in southern Mexico
Source: Parasit Vectors. 2015 Dec 21;8:651. doi: 10.1186/s13071-015-1266-7 (PMC4687067; doi:10.1186/s13071-015-1266-7)
Supplement: Additional file 7: — Natural selection and minimal number of recombination events for P. vivax merozoite proteins from different geographic origins. (DOCX 47 kb) [file 13071_2015_1266_MOESM7_ESM.docx]

**Additional file 7** Natural selection and minimal number of recombination events for *P. vivax* merozoite proteins from different geographic origins.

| Origin | | n | Tajima´s D | | |  | | |  | | | | | |  | | | *Rm* | |  |
| --- | --- | --- | --- | --- | --- | --- | --- | --- | --- | --- | --- | --- | --- | --- | --- | --- | --- | --- | --- | --- |
|  |  |  |  |  |  | Synonymous | | | Non-synonymous | | | | *dN-dS* | | | *P* | |  | | Sequence Reference: |
|  | *Gene fragment of 981pb: msp1_42_* | | | | | | | | | | | | | | | | | | | |
| CAM | | 44 | | 2.011 ns1 | | 7 | | 43 | | 2.254 | | | | | *0.013** | | | 12 | Parobek et al., 2014 | |
| SNG | | 50 | | -0.4728 ns2 | | 13 | | 67 | | 2.712 | | | | | *0.004** | | | 12 | Lee-Ching *et al.,* 2010 | |
| THAI “GQ” | | 58 | | 1.9206 ns1 | | 3 | | 44 | | 1.784 | | | | | 0.038* | | | 11 | Jongwutiwes et *al.,* 2010 | |
| THAI “AF” | | 35 | | 1.9511 ns1 | | 4 | | 43 | | 2.395 | | | | | 0.009* | | | 12 | Putaporntip *et al., 2000;*2002 | |
| THAI | | 93 | | 2.6745  P < 0.05 | | 4 | | 45 | | 2.153 | | | | | 0.017* | | | 12 | ** | |
| SLK | | 106 | | 2.5109  P < 0.05 | | 9 | | 48 | | 1.505 | | | | | 0.067* | | | 23 | Dias et al., 2011 | |
| SK 12 AÑOS | | 149 | | 2.888  P < 0.01 | | 3 | | 47 | | 4.121 | | | | | 0.000* | | | 4 | Kang *et al.,* 2012HANG 2012 | |
| TUR | | 30 | | 0.2083 ns2 | | 5 | | 22 | | 0.395 | | | | | 0.347 | | | 0 | Yildiz *et al.,* 2010 | |
| IND | | 30 | | 1.475 ns2 | | 8 | | 44 | | 1.678 | | | | | 0.048* | | | 13 | Putaporntip *et al., 2000;*2002 | |
| MYN | | 28 | | 1.651 ns2 | | 7 | | 44 | | 2.274 | | | | | 0.012* | | | 13 | Zhou and Chen, unpublished | |
| SK | | 51 | | 0.0915 ns2 | | 3 | | 41 | | 2.742 | | | | | 0.004* | | | 2 |  | |
| BRZ | | 11 | | 0.6307 ns2 | | 5 | | 40 | | 0.967 | | | | | 0.168 | | | 10 | Putaporntip *et al., 2000* | |
| *gene fragment of 780pb: ama1_I-II_* | | | | | | | | | | | | | | | | | | | | |
| IND | | 28 | | 0.3693 ns2 | 4 | | 25 | | | | | 1.788 | | 0.038* | | | 6 | | Rajesh et al., 2007 | |
| IND | | 32 | | 0.1054 ns2 | 8 | | 26 | | | | 1.502 | | | | *0.068* | | | 8 | | Thakur *et al.,* 2008 |
| IND | | 60 | | 0.3036 ns2 | 8 | | 28 | | | | 1.691 | | | | *0.047** | | | 9 | |  |
| IRN | | 83 | | 0.3923 ns2 | 4 | | 25 | | | | 1.443 | | | | *0.076* | | | 11 | | Zakeri et al., 2013 |
| IRN JX | | 37 | | 0.5402 ns2 | 4 | | 26 | | | | 1.364 | | | | 0.088 | | | 8 | | Zakeri et al., 2013 |
| IRN KF | | 46 | | 0.1091 ns2 | 4 | | 24 | | | | 1.55 | | | | 0.062 | | | 8 | | Zakeri et al., 2013 |
| PNG | | 102 | | 0.4580 ns2 | 3 | | 25 | | | | 2.58 | | | | *0.006** | | | 6 | | Arnott et al., 2013 |
| SLK | | 28 | | 0.4746 ns2 | 2 | | 24 | | | | 2.521 | | | | *0.007** | | | 6 | | Gunasekera *et al,* 2007 |
| THL | | 231 | | 0.5886 ns2 | 8 | | 26 | | | | 0.368 | | | | *0.357* | | | 11 | | Putaporntip *et al.,* 2009 |
| VNZ | | 73 | | 1.8032 ns1 | 2 | | 14 | | | | 1.822 | | | | *0.035** | | | 6 | | Ord *et al.,* 2008 |
| *Gene fragment of 663pb: dbp_II_* | | | | | | | | | | | | | | | | | | | | |
| COL | | 17 | | 1.133 ns2 | 0 | | 16 | | | | 3.922 | | | | *0.000* | | | 6 | | Ampudia *et al.,* 1996 |
| BRZ | | 122 | | 1.344 | 3 | | 17 | | | | 1.512 | | | | *0.067* | | | 6 | | Sousa *et al.,* 2010 |
| MYN | | 54 | | 0.4796 | 3 | | 24 | | | | 2.387 | | | | *0.009* | | | 7 | | Hyen-Lim *et al.,* 2012 |
| IND | | 95 | | -0.851 | 5 | | 33 | | | | 2.413 | | | | *0.009* | | | 10 | | Lim *et al.,* 2005; Prajapati *et al.,* 2008 |
| SLK | | 100 | | 0.764 | 4 | | 20 | | | | 1.227 | | | | *0.111* | | | 9 | | Premaratne *et al.,* 2009 |
| SK | | 111 | | -2.246  P < 0.01 | 14 | | 50 | | | |  | | | |  | | | 6 | | Kho *et al.,* 2001; Hye-Lim *et al.,* 2013; Lim C *et al.,* 2000, 2005 |
| THL | | 30 | | -0.772 | 4 | | 32 | | | | 1.999 | | | | *0.024* | | | 7 | | Gosi *et al.,* 2008 |
| IRN | | 130 | | 0.6979 | 2 | | 17 | | | | 1.512 | | | | *0.067* | | | 5 | | Babaeekho *et al.,* 2009; Valizadeh et al., 2014 |
| PNG | | 201 | | -1.434 | 15 | | 54 | | | | 2.33 | | | | *0.011* | | | 9 | | Xainli *et al.,* 2000; Cole-Tobian *et al.,* 2002,2005 |

Ns1, p> 0.05; Ns2, p>0.1
